# Maternal Hypertensive Disorders of Pregnancy and Offspring Risk of Hypertension: A Population-Based Cohort and Sibling Study

**DOI:** 10.1093/ajh/hpy176

**Published:** 2018-11-24

**Authors:** Azra Kurbasic, Abigail Fraser, Ingrid Mogren, Göran Hallmans, Paul W Franks, Janet W Rich-Edwards, Simon Timpka

**Affiliations:** 1 Department of Clinical Sciences Malmö, Genetic and Molecular Epidemiology Unit, Lund University Diabetes Centre, Clinical Sciences Malmö, Lund University, Malmö, Sweden; 2 Department of Population Health Sciences, Bristol Medical School, University of Bristol, Bristol, UK; 3 MRC Integrative Epidemiology Unit at the University of Bristol, University of Bristol, Bristol, UK; 4 NIHR Biomedical Research Centre at the University Hospitals Bristol NHS Foundation Trust and the University of Bristol, Bristol UK; 5 Department of Clinical Sciences, Obstetrics and Gynecology, Umeå University, Umeå, Sweden; 6 Department of Public Health and Clinical Medicine, Nutritional Research, Umeå University, Umeå, Sweden; 7 Department of Nutrition, Harvard T.H. Chan School of Public Health, Boston, Massachusetts, USA; 8 Department of Epidemiology, Harvard T.H. Chan School of Public Health, Boston, Massachusetts, USA; 9 Connors Center for Women’s Health and Gender Biology, Brigham and Women’s Hospital and Harvard Medical School, Boston, Massachusetts, USA

**Keywords:** blood pressure, cardiovascular diseases, epidemiologic studies, hypertension, “Hypertension, Pregnancy-Induced”, pre-eclampsia, pregnancy, preventive medicine

## Abstract

**BACKGROUND:**

Women with a history of hypertensive disorders of pregnancy (HDP) are at increased risk of hypertension, cardiovascular disease, and type 2 diabetes. Offspring from pregnancies complicated by HDP also have worse cardiometabolic status in childhood and young adulthood, but the offspring risk of clinical hypertension in adulthood is largely unknown.

**METHODS:**

We studied 13,893 first-born adult offspring (49.4% female) who attended a structured population-based primary care visit (The Västerbotten Health Survey) at age 40 years in Sweden between 1994 and 2013. Data on maternal HDP were collected from a population-based birth register. We investigated the association between maternal HDP and the risk of adult offspring hypertension and worse cardiometabolic risk factor status utilizing multivariable poisson and linear regression models. We also conducted a sibling comparison, which inherently accounted for familial factors shared by siblings (*N* = 135).

**RESULTS:**

Offspring participants of women with HDP (*N* = 383, 2.8%) had increased relative risk of hypertension (1.67, 95% confidence interval: 1.38, 2.01) and also higher mean body mass index, systolic blood pressure, diastolic blood pressure, and worse 2-hour 75 g oral glucose tolerance test result at age 40 years. No difference was observed for serum cholesterol. Point estimates for the cardiometabolic risk factors were attenuated in the sibling analyses.

**CONCLUSION:**

Offspring born to mothers with a history of HDP are on an adverse cardiometabolic trajectory and should be considered as concomitant targets for primordial prevention of hypertension in the maternal post-pregnancy period.

## INTRODUCTION

Hypertensive disorders of pregnancy (HDP; including gestational hypertension and pre-eclampsia) are serious complications during pregnancy, and women affected have higher risk of post-pregnancy cardiometabolic disease.^1^ Furthermore, the children born to these affected women and exposed to maternal HDP also have higher blood pressure^2^ and body mass index (BMI)^3^ compared with children born to normotensive mothers. These differences persist in young adulthood^4^ and might even translate into an increased risk of type 2 diabetes^5^ and stroke^6^ later in life. Results from animal models suggest that reduced uterine perfusion and exposure to anti-angiogenic factors during gestation—mimicking pre-eclampsia—can result in higher long-term blood pressure in the offspring, potentially mediated through altered cardiovascular structure and function.^7^ Yet, a sibling analysis of young adult offspring suggested that the association between maternal HDP and offspring cardiometabolic risk factors might be largely attributable to shared familial factors.^4^ However, no study has presented clinically measured hypertension data on middle-aged adults born to hypertensive or preeclamptic women and also included analysis of non-exposed siblings.^8^

In this study, we investigated the association between exposure to maternal HDP and clinically measured offspring hypertension and cardiometabolic risk factors at age 40 years. In a complementary sibling analysis, we attempted to determine the extent to which any associations reflected a shared propensity for more adverse cardiometabolic health.

## METHODS

We utilized data from standardized population-based preventive care visits in primary care at age 40 years (Västerbotten Health Survey)^9,10^ and comprehensive population-based registries of deliveries.^11^ Additional data on education and place of residence were collected via Statistics Sweden. The study was approved by the Regional Ethical Review Board at Lund University (2014/337).

### Study sample

We used data on first-born individuals who were born between 1955 and 1972 in the counties covered by the comprehensive birth register (described below) and who attended the Västerbotten Health Survey at age 40 years between 1994 and 2013. In total, 14,947 first-born participants were initially eligible; we further excluded those outside the permitted age-range or with missing data on co-variables used in the adjusted analyses (Supplementary Figure 1). As there were small amounts of missing outcome data, the number of participants included in each analysis varied slightly.

### Data on maternal HDP

Maternal HDP was ascertained using data from a comprehensive regional birth register covering 2 counties in northern Sweden. This resource has previously been used for epidemiological research.^11,12^ Maternal HDP (pre-eclampsia/toxemia/eclampsia/gestational hypertension) and any diabetes mellitus during pregnancy were defined according to the International Classification of Diseases (Supplementary Material). No specific data on pre-pregnancy hypertension status was available.

### Clinical visits at age 40 years

At age 40 years, all residents of the county of Västerbotten were invited to a standardized visit in primary care focused on cardiometabolic disease prevention. Approximately, 60% of the eligible population attended.^10^ The standardized study visits were focused on lifestyle, are performed by a nurse, and include a self-administered questionnaire on previous diagnoses, smoking habits, and family history of cardiovascular disease. Body weight and height were measured, and we calculated BMI as kg/m^2^. Systolic blood pressure (SBP) and diastolic blood pressure (DBP) were measured once with a sphygmomanometer with the participant in a supine position before August 2009 and subsequently twice in a seated position (average values used). For analytic purpose, we converted seated blood pressure measurements to supine equivalents using previously published age- and sex-specific equations based on data from the same setting (Supplementary Material).^13^ Hypertension was defined either through self-report, current anti-hypertensive treatment, or clinical measurement of SBP ≥140 and/or DBP ≥90 mm Hg.

The visit also included a 75 g 2-hour oral glucose tolerance test (OGTT) with capillary sampling. Total serum cholesterol was measured using Reflotron bench-top analyzers before 2009 and was analyzed at the regional clinical chemistry lab (at University Hospital of Umeå).

### Statistical analysis

First, we investigated the association between maternal HDP and offspring relative risk of hypertension at age 40 years. To do so, we used Poisson regression models incrementally adjusted for co-variables. Model I included maternal HDP in index pregnancy, whereas model II (main model) additionally included offspring sex, family history of cardiovascular disease, and maternal diabetes during pregnancy. In model III, we further included education level, smoking, and BMI at age 40 years (when BMI was not the outcome).

We then investigated the association between maternal HDP and offspring BMI, SBP, DBP, 2-hour OGTT result, and log-transformed total serum cholesterol, respectively. To do this, we utilized linear regression models incrementally adjusted for co-variables as above. The use of anti-hypertensive or lipid-lowering medication was accounted for by including constants as previously recommended (Supplementary Material).^14,15^

For the sibling analyses, we established a second cohort by identifying siblings who attended the age 40 years visit (*N* = 62 sibships) with at least 1 sibling in each sibship being exposed to maternal HDP. We compared cardiometabolic risk factors in the sibling cohort by a linear mixed model with random family effects to account for multiple offspring by the same mother. We adjusted also these analytical models incrementally for co-variables. All statistical analyses were performed in SAS 9.3 (SAS Institute, Cary, NC).

## RESULTS

Of the total study sample (*N* = 13,893), 383 participants (2.8%) were exposed to maternal HDP. At age 40 years, there were no substantial differences in smoking status or education level between participants exposed and not exposed to maternal HDP. Supplementary Table 1 shows the study sample characteristics.

Participants exposed to maternal HDP had a higher relative risk of hypertension in model I (1.70, 95% confidence interval (CI): 1.41, 2.04), model II (1.67, 95% CI: 1.38, 2.01), and in model III (1.52, 95% CI: 1.27, 1.84).

Table 1 shows the associations between maternal HDP and continuous cardiometabolic risk factors in exposed offspring at age 40 years. Exposure to maternal HDP was associated with higher mean BMI, SBP, DBP, and 2-hour OGTT result in model II. There was no evidence of an interaction between exposure to maternal HDP and offspring sex when analyzing the continuous outcomes (*P* > 0.05, data not shown). Results remained similar in analyses accounting for medication with dummy variables (data not shown).

**Table 1.** Cardiometabolic risk factors at age 40 years in offspring exposed to maternal HDP

	Mean difference^a^	95% CI	*P*
BMI, kg/m^2^			
Model I	0.88	0.43, 1.32	<0.001
Model II	0.84	0.40, 1.28	<0.001
Model III	0.82	0.38, 1.26	<0.001
SBP, mm Hg			
Model I	4.89	3.46, 6.31	<0.001
Model II	4.72	3.35, 6.09	<0.001
Model III	3.86	2.56, 5.15	<0.001
DBP, mm Hg			
Model I	3.04	2.01, 4.07	<0.001
Model II	2.92	1.92, 3.93	<0.001
Model III	2.29	1.34, 3.24	<0.001
2-hour OGTT glucose, mmol/l			
Model I	0.13	−0.01, 0.27	0.06
Model II	0.14	0.01, 0.28	0.04
Model III	0.10	−0.03, 0.24	0.13
Log total serum cholesterol^b^			
Model I	−0.01	−0.03, −0.01	0.27
Model II	−0.01	−0.03, −0.01	0.20
Model III	−0.02	−0.04, 0.00	0.11

Model I only includes exposure to maternal HDP (yes or no).

Model II additionally includes offspring sex (female or male), family history of cardiovascular disease (yes or no), and maternal diabetes mellitus during pregnancy (yes or no).

Model III additionally includes body mass index (when it is not the outcome), smoking (yes or no), and education (9 years or less, 10–12 years, 12 years or more).

^a^All mean differences compared with offspring not exposed to maternal HDP.

^b^Results interpreted as % (×100) difference in mean.

Abbreviations: BMI, body mass index; CI, confidence interval; DBP, diastolic blood pressure; HDP, hypertensive disorders of pregnancy; OGTT, oral glucose tolerance test; SBP, systolic blood pressure.

Supplementary Table 2 shows the study characteristics of the study sample in the sibling analysis by exposure to maternal HDP. In total, 135 participants were included. There was little evidence of associations of exposure to HDP with worse cardiometabolic risk factor profile in siblings, with more modest point estimates, i.e., closer to the zero, than those found in the population overall. In model II, the association for BMI was −0.28 kg/m^2^ (95% CI: −1.66, 1.09), for SBP −0.06 mm Hg (95% CI: −4.36, 4.24), for DBP 0.004 mm Hg (95% CI: −2.96, 2.96), for 2-hour OGTT glucose 0.07 mmol/l (95% CI: −0.37, 0.51) and for total serum cholesterol 4 % (95% CI: −2%, 9%) (Supplementary Table 3).

## DISCUSSION

In this study, we for the first time report offspring exposed to maternal HDP during pregnancy to have higher risk of hypertension based on clinical measurements in middle age. Furthermore, we found that exposure to maternal HDP is associated with higher adult blood pressure and BMI. In contrast, we found less evidence of a difference in cardiometabolic health between siblings with differential fetal HDP exposure.

Thoulass et al.^8^ found few studies of adult offspring by exposure to maternal HDP and cardiometabolic outcomes. Nevertheless, offspring born to women with pregnancies complicated by HDP appeared to have slightly higher blood pressure, higher risk of hypertension, and higher BMI compared with offspring from uncomplicated pregnancies. Our study includes a definition of hypertension based on clinical measurement of blood pressure, confirming an association between maternal HDP and offspring hypertension in middle age that was previously suggested by self-report and administrative health care data. In this study, we also report a worse OGTT result in participants exposed to maternal HDP, which was partly explained by higher BMI. Kajantie et al.^5^ have previously reported offspring exposed to maternal hypertension in pregnancy to have increased risk of type 2 diabetes, defined as collecting prescriptions on diabetic medications in late middle age.

When analyzing siblings discordant in HDP exposure, we found no difference in cardiometabolic risk factors. Although the statistical power is lower in the restricted sample compared with the main analyses, the size of the effect estimates was also reduced. Alsnes et al.^4^ previously reported that young adults born to mothers with a history of hypertension in pregnancy had a more adverse cardiovascular profile, but, consistent with our findings, there was little difference between exposed and non-exposed siblings. This suggests that the association between exposure to maternal HDP and adverse cardiometabolic health might partly be explained by a familial propensity, which might have genetic, behavioral, or environmental origins.

The main strength of this study is the combination of cardiometabolic outcomes collected in a standardized clinical setting, the large population-based sample, and the outcome registration in early middle age. During the period relevant for this study, Sweden had already implemented universal maternity care, so the threshold to access care for symptomatic women should have been low. Still, the small proportion of offspring exposed to maternal HDP was expected,^16^ and we have, therefore, not analyzed outcomes by type of HDP. Moreover, at the time of the maternal pregnancies in the 1950s to 1970s, the distinction between gestational hypertension and pre-eclampsia was not yet as distinct as in the contemporary clinical setting. We have focused on the ICD diagnoses that we deem most likely to signify severe clinical disease, but the study is limited by specific data on maternal pre-pregnancy hypertension not being available. As we use a regional register to collect data on pregnancy-related diagnoses, our sample is limited to those born in the same part of Sweden and who also attended the standardized clinical visits. We do not expect this to induce any major bias, as we do not think relocating before middle age has any strong implications for the association between exposure to maternal HDP and cardiometabolic risk factors in middle age.

Ascertaining exposure to maternal HDP in adult offspring in a clinical setting is not feasible. However, our results suggest that the children of women with a history of HDP should be considered concomitant targets of prevention. It has previously been suggested that family interventions targeting cardiometabolic health might be suitable in women with a history of HDP.^17^ As differences were largely attenuated when we accounted for familial factors, this study supports targeting all siblings and not only those with direct fetal exposure to HDP.

To summarize, we report an association between maternal HDP and worse cardiometabolic status at age 40 years in exposed offspring, including a 67% increased risk of hypertension. Further analyses suggested that these associations might be explained by shared familial factors. Regardless, interventions targeting women with a history of HDP to promote cardiometabolic health post-pregnancy should also consider their children, perhaps through family-based approaches.

## DISCLOSURE

A.K. is currently employed at Leo Pharma A/S, Copenhagen, Denmark.

## Supplementary Material

Supplementary Material
