# Supplementary material for: Maternal Hypertensive Disorders of Pregnancy and Offspring Risk of Hypertension: A Population-Based Cohort and Sibling Study
Source: Am J Hypertens. 2018 Nov 24;32(4):331–4. doi: 10.1093/ajh/hpy176 (PMC6420682; doi:10.1093/ajh/hpy176)
Supplement: Supplementary Material [file hpy176_suppl_supplementary_material.docx]

**ONLINE SUPPLEMENT**

**American Journal of Hypertension**

**Maternal hypertensive disorders of pregnancy and offspring risk of hypertension: a population-based cohort and sibling study**

Kurbasic et al.

**Methods supplement**

For the purpose of correcting for current medication when analyzing continuous outcomes, adding a sensible constant appears to be better than including a dummy variable in the regression model or not making any adjustment at all.^1^ To adjust for the use of medication, we therefore used constants added to the clinical measurement when analyzing outcomes in linear models. Systolic blood pressure (SBP) and diastolic blood pressure (DBP) measurements were corrected for the use of antihypertensive medication (SBP +15 mmHg and DBP +10 mmHg, respectively).^1^ Total serum cholesterol values were corrected for the use of lipid-lowering medication (assumed to be statins) using a published constant (+1.347 mmol/l).^2^

Maternal pregnancy data

We have utilized data from a regional birth register in Northern Sweden.^3^ We defined hypertensive disorders of pregnancy (HDP) according to the international classification of disease (ICD) 8 (Swedish version). The following specific sub-codes for the codes 637 (*Pre-eclampsia, eclampsia and toxaemia, unspecified*) and 661 (*Delivery with other complications*) were used: 637.01 (*Hypertonia*); 637.03 (*Prae-eclampsia levis*); 637.04 (*Prae-eclampsia gravis*); 637.09 (*Prae-eclampsia non-ultra descriptus*); 637.10 (*Eclampsia*); 661.2 (*Eclampsia (prae-eclampsia) intra partum*). We defined diabetes mellitus as ICD-8 code 250 (*Diabetes mellitus*).

Conversion of blood pressure measurements

It has previously been shown that the difference between seated and supine blood pressure is sex- and age-dependent but independent of body mass index (BMI).^4^ To convert seated blood pressure measurements to supine measurements, we utilized previously published sex- and age-specific equations developed in the same clinical setting as that in which the study data were collected.^4^ In short, blood pressure was measured twice in both supine and seated position in 210 40-year old participants (50% women) recruited from both rural and urban primary care centers in the county. These measurements were then used to develop equations to convert seated blood pressure to supine and vice versa. To account for the change of measurement procedure in 2009, for men we calculated supine SBP as 24.595+(0.792 x seated SBP) and supine DBP as 17.282+(0.753 x seated DBP). For women the corresponding equations were 8.669+(0.919 x seated SBP) and 5.784+(0.890 x seated DBP).

Power calculation

A simplified power calculation *post hoc* (not accounting for correlation between siblings) suggested that there was 80% power to detect a 6 mmHg difference in systolic blood pressure, assuming a mean value of 125 (15 SD) mmHg in the comparison group. For this calculation, we further assumed a sample size of 200 of which 95 participants had been exposed to maternal HDP and alpha=0.05. For the purpose of this study, we assessed the results and sample size in the previous study by Alsnes *et al.*^6^ to provide a reasonable assurance that the reported analyses were worthwhile in the available dataset.

**Results supplement**

In crude analyses for which we did not make any adjustment for blood pressure medication or lipid lowering drugs, respectively, the estimates for blood pressure and cholesterol (Supplemental Table 4) were very similar to the results of Model I in our main analyses.

**Supplemental references**

1. Tobin MD, Sheehan NA, Scurrah KJ, Burton PR. Adjusting for treatment effects in studies of quantitative traits: antihypertensive therapy and systolic blood pressure. *Stat Med*. 2005; 24:2911-2935. <https://doi.org/10.1002/sim.2165>.

2. Wu J, Province MA, Coon H, Hunt SC, Eckfeldt JH, Arnett DK, Heiss G, Lewis CE, Ellison RC, Rao DC, Rice T, Kraja AT. An investigation of the effects of lipid-lowering medications: genome-wide linkage analysis of lipids in the HyperGEN study. *BMC Genet*. 2007; 8:60. <https://doi.org/10.1186/1471-2156-8-60>.

3. Sandström A, Nyström L. Uppbyggnad av ett medicinskt födelseregister för perioden 1955–1972. *Socialmedicinsk Tidskr*. 1985:95 – 7.

4. Cicolini G, Pizzi C, Palma E, Bucci M, Schioppa F, Mezzetti A, Manzoli L. Differences in Blood Pressure by Body Position (Supine, Fowler’s, and Sitting) in Hypertensive Subjects. *Am J Hypertens*. 2011; 24:1073-1079. <https://doi.org/10.1038/ajh.2011.106>.

5. Ng N, Carlberg B, Weinehall L, Norberg M. Trends of blood pressure levels and management in Västerbotten County, Sweden, during 1990–2010. *Glob Health Action*. 2012; 5. <https://doi.org/10.3402/gha.v5i0.18195>.

6. Alsnes IV, Vatten LJ, Fraser A, Bjørngaard JH, Rich-Edwards J, Romundstad PR, Åsvold BO. Hypertension in Pregnancy and Offspring Cardiovascular Risk in Young Adulthood: Prospective and Sibling Studies in the HUNT Study (Nord-Trøndelag Health Study) in Norway. *Hypertens Dallas Tex 1979*. 2017; 69:591-598. <https://doi.org/10.1161/HYPERTENSIONAHA.116.08414>.

**Supplemental Tables and Figures**

**Supplemental Table 1.** Study sample characteristics by maternal hypertensive disorders of pregnancy

**Supplemental Table 2.** Descriptive characteristics of the study sample in sibling analysis by maternal hypertensive disorders of pregnancy (N=135)

**Supplemental Table 3.** The difference in cardiometabolic risk factors in siblings with discordant exposure to maternal hypertensive disorders of pregnancy

**Supplemental Table 4.** The crude unadjusted associations between offspring exposure to maternal hypertensive disorders of pregnancy and cardiometabolic status

**Supplemental Figure 1.** Flow chart of study sample identification

| **Supplemental Table 1**. Study sample characteristics by maternal hypertensive disorders of pregnancy | | | | |  |
| --- | --- | --- | --- | --- | --- |
|  | No maternal HDP |  | Maternal HDP |  | |
|  |  | Missing data, N |  | Missing data, N | |
| Participants, N (%) | 13,510 (97.2 ) | - | 383 (2.8) | - | |
| Female, N (%) | 6,680 (49.4) | - | 187 (48.9) | - | |
| Maternal diabetes mellitus during pregnancy | 28 (0.21) | - | 1 (0.26) | - | |
| Age at age 40 visit, years (SD) | 40.7 (0.2) | - | 40.1 (0.2) | - | |
| Education level, N (%) |  | - |  | - | |
| - 9 years or less | 819 (6.1) |  | 25 (6.5) |  | |
| - 10 to 12 years | 7,280 (53.9) |  | 211 (55.1) |  | |
| - 12 years or more | 5,411 (40.1) |  | 147 (38.4) |  | |
| Family history of CVD, N (%) | 2,403 (17.8) | - | 76 (19.8) | - | |
| Smoking, N (%) | 1,738 (12.9) | - | 42 (11.0) | - | |
| SBP, Mean (SD) mmHg | 120 (14) | 47 | 125 (16) | 2 | |
| DBP, Mean (SD) mmHg | 76 (10) | 53 | 79 (11) | 2 | |
| Hypertension, N (%) | 1,876 (13.9) | - | 90 (23.8) | - | |
| BMI, Mean (SD) kg/m^2^ | 25.8 (4.4) | - | 26.7 (4.6) | - | |
| 2h OGTT capillary glucose, Mean (mmol/L) | 6.4 (1.4) | 428 | 6.5 (1.3) | 10 | |
| Total serum cholesterol, Median (IQR) mmol/l | 5.10 (4.49, 5.80) | 45 | 4.98 (4.40, 5.73) | 1 | |
| BMI: Body mass index; CVD: Cardiovascular disease; DBP: Diastolic blood pressure; HDP: Hypertensive disorders of pregnancy; IQR: Interquartile range; OGTT: Oral glucose tolerance test; SBP: Systolic blood pressure; SD: Standard deviation | | | | |  |

| **Supplemental Table 2**. Descriptive characteristics of the study sample in sibling analysis by maternal hypertensive disorders of pregnancy (N=135) | | | | |  |
| --- | --- | --- | --- | --- | --- |
|  | No maternal HDP |  | Maternal HDP |  | |
|  |  | Missing data, N |  | Missing data, N | |
| Participants, N (%) | 70 (51.8) | - | 65 (48.2) | - | |
| Female, N (%) | 34 (48.6) | - | 34 (52.3) | - | |
| Age at age 40 visit, years (SD) | 40.1 (0.2) | - | 40.1 (0.2) | - | |
| Education level, N (%) |  | - |  | - | |
| - 9 years or less | 2 (2.9) |  | 4 (6.1) |  | |
| - 10 to 12 years | 42 (65.7) |  | 33 (50.8) |  | |
| - 12 years or more | 22 (31.4) |  | 28 (43.1) |  | |
| Family history of CVD, N (%) | 6 (8.6) | - | 13 (20.0) | - | |
| Smoking, N (%) | 5 (7.1) | - | 3 (3.6) | - | |
| SBP, Mean (SD) mm/Hg | 124 (16) | - | 125 (14) | 1 | |
| DBP, Mean (SD) mm/Hg | 78 (12) | - | 79 (12) | 1 | |
| BMI, Mean (SD) kg/m^2^ | 26.1 (4.7) | - | 25.8 (4.0) | - | |
| 2h OGTT capillary glucose, Mean (mmol/L) | 6.4 (1.2) | 1 | 6.4 (1.3) | 1 | |
| Total serum cholesterol, Median (IQR) mmol/l | 5.02 (1.2) | - | 5.23 (1.1) | - | |
| BMI: Body mass index; CVD: Cardiovascular disease; DBP: Diastolic blood pressure; HDP: Hypertensive disorders of pregnancy; IQR: Interquartile range; OGTT: Oral glucose tolerance test; SBP: Systolic blood pressure; SD: Standard deviation | | | | |  |

| **Supplemental Table 3.** The difference in cardiometabolic risk factors in siblings with discordant exposure to maternal hypertensive disorders of pregnancy | | | |
| --- | --- | --- | --- |
|  | Mean difference | 95% CI | *P* |
| BMI, kg/m^2^ (N=135, NS=62) |  | |  |
| Model I | -0.22 | -1.57, 1.14 | 0.75 |
| Model II | -0.28 | -1.66, 1.09 | 0.68 |
| Model III | -0.27 | -1.63, 1.10 | 0.70 |
| SBP, mmHg (N=133, NS=61) |  | |  |
| Model I | 0.10 | -4.11, 4.30 | 0.96 |
| Model II | -0.06 | -4.36, 4.24 | 0.98 |
| Model III | -0.12 | -4.45, 4.20 | 0.95 |
| DBP, mmHg (N=133, NS=61)) |  | |  |
| Model I | 0.39 | -2.71, 3.49 | 0.80 |
| Model II | 0.004 | -2.96, 2.96 | 0.99 |
| Model III | -0.08 | -3.10, 2.95 | 0.96 |
| 2h OGTT glucose, mmol/l (N=132, NS=61) |  | |  |
| Model I | 0.07 | -0.38, 0.51 | 0.76 |
| Model II | 0.07 | -0.37, 0.51 | 0.75 |
| Model III | 0.13 | -0.30, 0.56 | 0.55 |
| Log total serum cholesterol^a^ (N=135, NS=62) |  | |  |
| Model I | 0.04 | -0.01, 0.10 | 0.13 |
| Model II | 0.04 | -0.02, 0.09 | 0.19 |
| Model III | 0.04 | -0.01, 0.10 | 0.12 |
| BMI: Body mass index; CI: Confidence interval; Diastolic blood pressure; HDP: Hypertensive disorders of pregnancy; OGTT: Oral glucose tolerance test; NS: Number of sib-ships; SBP: Systolic blood pressure  **a** Results interpreted as % (x100) difference in mean  Model I only includes exposure to maternal HDP (yes or no)  Model II additionally includes offspring sex (female or male), reported family history of cardiovascular disease (yes or no)  Model III additionally includes body mass index (when it is not the outcome), smoking (yes or no) and education (9 years or less, 10-12 years, 12 years or more) | | | |

| **Supplemental Table 4.** The crude unadjusted associations between offspring exposure to maternal hypertensive disorders of pregnancy and cardiometabolic status | | | |
| --- | --- | --- | --- |
|  | Exposure to maternal HDP, mean difference | 95% CI | *P* |
| SBP, mmHg | 4.15 | 2.75, 5.54 | <0.001 |
| DBP, mmHg | 2.47 | 1.44, 3.49 | <0.001 |
| Log total serum cholesterol^a^ | -0.01 | -0.03, 0.01 | 0.24 |
| CI: Confidence interval; Diastolic blood pressure; HDP: Hypertensive disorders of pregnancy; SBP: Systolic blood pressure  No constants have been added to account for current medication or type of blood pressure examination.  **a** Results interpreted as % (x100) difference in mean | | | |


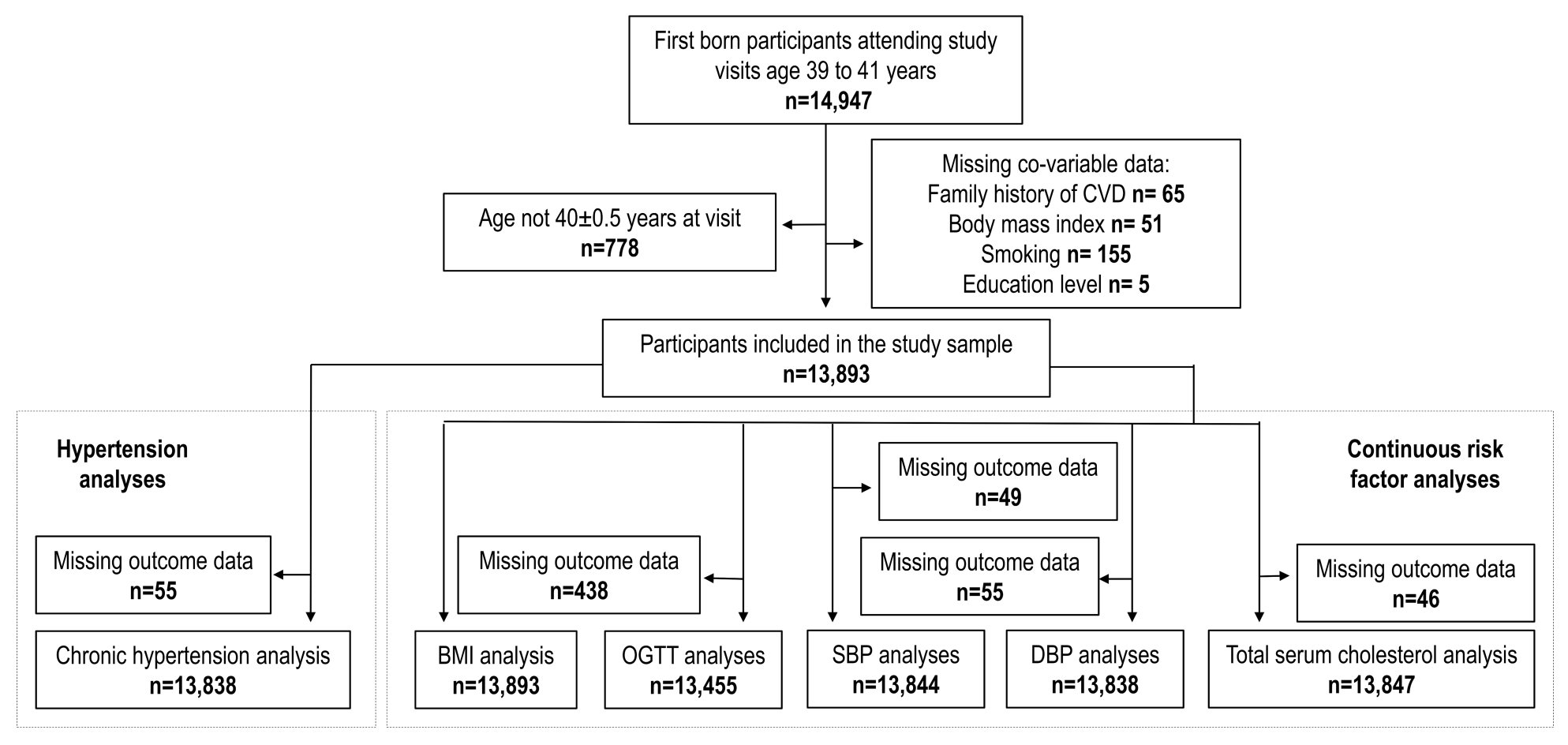


**Supplemental Figure 1**. Flow chart of study sample identification

**BMI:** Body mass index; **CVD:** Cardiovascular disease; **DBP:** Diastolic blood pressure; **OGTT:** Oral glucose tolerance test; **SBP:** Systolic blood pressure
